# A synonymous mutation of rs1137070 cause the mice *Maoa* gene transcription and translation to decrease

**DOI:** 10.3389/fnmol.2024.1406708

**Published:** 2024-09-18

**Authors:** Kai Xin Li, Lei Fan, Hongjuan Wang, Yushan Tian, Sen Zhang, Qingyuan Hu, Fanglin Liu, Huan Chen, Hongwei Hou

**Affiliations:** ^1^Division of Life Sciences and Medicine, University of Science and Technology of China, Hefei, China; ^2^China National Tobacco Quality Supervision and Test Center, Zhengzhou, China; ^3^Key Laboratory of Tobacco Biological Effects, Zhengzhou, China; ^4^Beijing Life Science Academy, Beijing, China; ^5^Institute of Technical Biology and Agricultural Engineering, Hefei Institutes of Physical Science, Chinese Academy of Sciences, Hefei, China; ^6^Department of Bioengineering, School of Chemical Engineering, Northwest University, Xi’an, China

**Keywords:** Maoa EcoRV polymorphism, Monoamine oxidase A, synonymous mutation, rs1137070, mouse model

## Abstract

The Monoamine Oxidase-A (*MAOA*) EcoRV polymorphism (rs1137070) is a unique synonymous mutation (c.1409 T > C) within the *MAOA* gene, which plays a crucial role in *Maoa* gene expression and function. This study aimed to explore the relationship between the mouse *Maoa* rs1137070 genotype and differences in *MAOA* gene expression. Mice carrying the CC genotype of rs1137070 exhibited a significantly lower *Maoa* expression level, with an odds ratio of 2.44 compared to the T carriers. Moreover, the wild-type TT genotype of MAOA demonstrated elevated mRNA expression and a longer half-life. We also delved into the significant expression and structural disparities among genotypes. Furthermore, it was evident that different aspartic acid synonymous codons within *Maoa* influenced both *MAOA* expression and enzyme activity, highlighting the association between rs1137070 and MAOA. To substantiate these findings, a dual-luciferase reporter assay confirmed that GAC was more efficient than GAT binding. Conversely, the synonymous mutation altered *Maoa* gene expression in individual mice. An RNA pull-down assay suggested that this alteration could impact the interaction with RNA-binding proteins. In summary, our results illustrate that synonymous mutations can indeed regulate the downregulation of gene expression, leading to changes in MAOA function and their potential association with neurological-related diseases.

## GRAPHICAL ABSTRACT

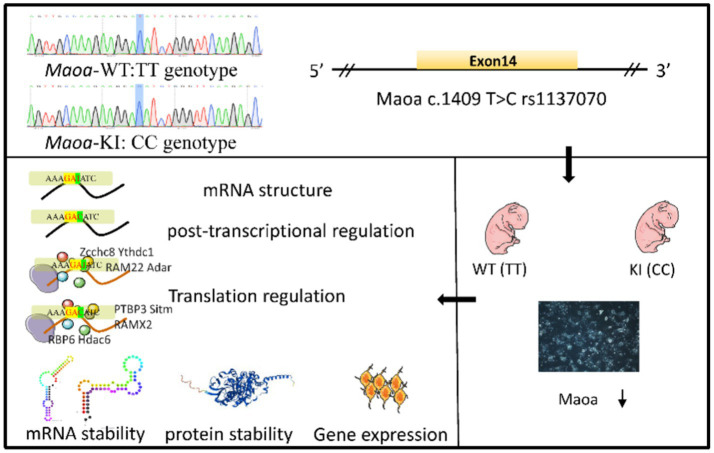

## Highlights

- From the perspective of epigenetics, the regulatory mechanism of synonymous mutations on MAOA activity and structure was explored.
- A mouse GAC gene mutation model was constructed to detect gene expression products after obtaining primary cells.
- GAC is a better codon than GAT, with higher translation efficiency but worse stability.
- Improving the genetic mutations in the genetic spectrum of the Chinese population is highly significant.

## Introduction

Monoamine Oxidase A (*MAOA*) is a metabolic enzyme responsible for the breakdown of neurotransmitters such as dopamine, norepinephrine, serotonin, and other monoamines. These monoamines are considered pivotal neurotransmitters in the pathophysiology of conditions like schizophrenia (Sun et al., 2012). MAOA has been consistently associated with conditions such as addiction, depression, autism, and antisocial behavior (Byrd and Manuck, 2014; Kolla and Bortolato, 2020). Additionally, MAOA plays a critical role in regulating neurotransmitters in the brain that are closely linked to emotions, including depression, reward, and aggression (Cases et al., 1995). Since the mid-1990s, researchers have been investigating the associations between genetic variants and various phenotypic abnormalities (Manuck et al., 2000). Among these polymorphisms, one of the primary focuses has been on rs1137070, which has been found to be associated with susceptibility to smoking (Shen et al., 2019), heroin addiction (Sun et al., 2017), hostility, and depression (Yen et al., 2021), as well as susceptibility to Parkinson’s disease (Sun et al., 2014), and autism (Verma et al., 2014), etc.

The rs1137070 is a C to T substitution at position 1,460 of the DNA sequence (c.1460C > T) in *Maoa* exon 14. This synonymous single nucleotide polymorphism (SNP) results in a synonymous change (Asp470Asp) and is located in the coding region of the X chromosome. A 2019 study found that polymorphisms in *Maoa* (rs1137070) play a moderating role in the effects of smoking (Shen et al., 2019). Other studies on rs1137070 demonstrated that individuals carrying the *Maoa* EcoRV polymorphism C allele (rs1137070; those with the 1460C > T polymorphism) in exon 14 had lower MAOA activity (Agatsuma et al., 2006). However, these studies on *Maoa* gene polymorphisms have primarily relied on questionnaires, brain imaging, and physiological and biochemical analyses, with the exact underlying mechanism remaining unclear. In addition, there is a lack of research on rs1137070 in cells and animals, so it is necessary to construct relevant cell and animal models to further complement the molecular mechanism of rs1137070 locus affecting MAOA.

Synonymous mutation refers to a mutation in which the codon is different but does not change the encoded amino acid. Increasing evidence indicates that synonymous codons are not used randomly or equally (Hanson and Coller, 2018). Concerning genetic polymorphisms of synonymous codons, studies have identified a common phenomenon known as codon usage bias (CUB), which involves the differential usage of synonymous codons of varying frequencies and is prevalent across various species (Liu et al., 2021b). It is now recognized that codon usage affects gene expression by regulating factors such as elongation speed, translation efficiency, initiation, termination, and accuracy. There have been relatively few animal experiments aimed at investigating the contribution of synonymous mutations to expression regulation or comprehensively assessing their impact on organisms. Therefore, despite the findings from population studies linking synonymous codon mutations to a variety of mood disorders, this paper aims to further elucidate the differential regulatory mechanisms of transcription and translation caused by synonymous mutations by constructing base-editing model animals.

The CRISPR/Cas9 system has gained widespread recognition for its utility in targeted insertion of exogenous DNA sequences, offering an effective method for creating animal models with synonymous mutations (Banan, 2020). In recent years, various genome editing tools have been developed to facilitate the creation of site-specific DNA double-strand breaks (DSBs). Examples include zinc finger nucleases (ZFNs), transcription activator-like effector nucleases (TALENs), and the clustered regularly spaced short palindromic repeats CRISPR-Cas9 systems (Gaj et al., 2013). Cas9 can bind to multiple single-guide RNAs (sgRNAs), enabling efficient multiplexed genome editing in mammalian cells. This technology has found extensive use in the development of animal models featuring synonymous mutations in genes (Albadri et al., 2017; Kim et al., 2020). Cas9-engineered mice have become valuable assets in biological and disease modeling (Platt et al., 2014).

Genome-wide association studies (GWAS) have revealed substantial differences in the frequency distribution of this gene among different racial populations (Sirugo et al., 2019). Therefore, the primary objective of this study is to unravel the impact of genetic diversity within the human *Maoa* gene on gene biosynthesis and function through the creation of synonymous mutations in mice *Maoa* at position 1,409 T > C. To achieve this, we employed the CRISPR-Cas system to introduce the 1,409 T > C mutation into the mouse *Maoa* gene, homozygous gene mice with KI were obtained. This research aims to investigate the dysfunctional and molecular regulatory mechanisms of single nucleotide polymorphisms (SNPs) by assessing MAOA protein expression. We also seek to uncover the underlying mechanisms through which this synonymous mutation influences mRNA and protein structure, as well as its impact on transcriptional translation regulation. In summary, our findings offer fresh insights into the role of synonymous mutations in MAOA in the regulation of gene expression, shedding light on a new perspective for understanding the mechanism of MAOA enzyme activity. This research holds significant implications for understanding the pathogenesis of this gene mutation in the population and contributes to enhancing our comprehension of genetic diversity within the Chinese population.

## Materials and methods

Key resources are presented in Table 1.

**Table 1:** Key resources.

Reagent or Resource	Source	Identifier
**Antibodies**		
Tyrosine Hydroxylase (E2L6M) Rabbit mAb	Cell Signaling Technology	Cat#58844; RRID:AB_2303165
Rabbit (DA1E) mAb IgG XP® isotype control (Alexa Fluor® 488 Conjugate)	Cell Signaling Technology	Cat2975S; RRID:AB_1196589
MAOA (E3L3B) Rabbit mAb	Cell Signaling Technology	Cat#75330
GAPDH (D16H11) XP® Rabbit mAb	Cell Signaling Technology	Cat#5174 T; RRID:AB_10622025
Anti-rabbit IgG (H + L), F(ab’)2 Fragment (Alexa Fluor® 555 Conjugate)	Cell Signaling Technology	Cat#4413S, RRID:AB_10694110
Anti-rabbit IgG, HRP-linked antibody	Cell Signaling Technology	Cat#7074, RRID:AB_2099233
**Cell**		
293 T	National Collection of Authenticated Cell Cultures	Cat#GNHu17
**Experimental models**		
Mouse: C57BL/6 J	Beijing Vital River Laboratory Animal Technology Co., Ltd. (China)	N/A
Mouse:C57BL/6 J-Maoaem1Cin(T1410C)	Gempharmatech Co., Ltd	N/A
Plasmids and viruses	Henan Writegene Biotechnology CO., LTD	N/A
Rluc sea nephrophilase wild/mutant plasmid	Henan Writegene Biotechnology CO., LTD	N/A
psiCheck2	Henan Writegene Biotechnology CO., LTD	N/A
**Chemicals**		
Dual-Luciferase reporter assay	Beyotime Biotechnology	Cat#RG027
MAO-Glo™ ASSAY	Promage	Cat#V1402
Mouse Monoamine oxidase A (MAOA) ELISA assay kit	Jianglai biology	Cat#JL14083
Actinomycin D	MedChemExpress	Cat#HY-17559
Cycloheximide	MedChemExpress	Cat# HY-12320
**Software and algorithms**		
GraphPad Prism 9	GraphPad	https://www.graphpad.com/
RNA Folding Form V2.3	The UNAFold Web Server	http://www.unafold.org/mfold
AlphaFold protein structure database	EMBL-EBI	https://www.alphafold.ebi.ac.uk/

## Animals and ethical statement

Six-week-old C57BL/6 J *Maoa*-KI mice (synonymous mutations in mouse MAOA at position 1,409 T > C) were acquired from Gempharmatech Co., Ltd. *Maoa*-WT mice were acquired from Beijing Vital River Laboratory Animal Technology Co., Ltd. Both C57 Mouse were obtained and used as both embryo donors and foster strains. All experimental procedures were conducted in compliance with the protocols approved by the Laboratory Animal Management and Ethics Committee of China National Tobacco Quality Supervision and Test Center. These procedures adhered to established experimental practices and standards, and All animal experiments are comply with the ARRIVE guidelines and should be carried out in accordance with the U.K. Animals (Scientific Procedures) Act, 1986 and associated guidelines, EU Directive 2010/63/EU for animal experiments, or the National Research Council’s Guide for the Care and Use of Laboratory Animals. Mice were housed and bred in a specific pathogen-free (SPF) animal facility, with five mice per cage to minimize the occurrence of aggression and injuries. They were allowed to acclimate for 1 week before the commencement of experiments, during which they had free access to both food and water. The light and dark cycles were maintained at 12 h each, with a constant temperature of 25°C and humidity ranging between 40 and 60% in the facility. Mice were randomly assigned to different experimental groups. Mice were subjected to cervical dislocation for NSC collection Sampling was conducted between 9:00 and 15:00 from August to January (Kasowitz et al., 2018; Gupta et al., 2019; Liu et al., 2021a).

### Experimental model

#### Cell culture

Human Embryonic Kidney 293 T (293 T) cells and Male Mouse Neuron Primary Cells were utilized in this study. The original HEK293 cell line is derived from a deceased human embryonic kidney cell. HEK293 cells are created by introducing human adenovirus type 5 DNA fragments into human embryonic kidney cells. The 293 T cell line is a highly transfectable derivative of the HEK293 cell line, into which the temperature-sensitive gene for SV40 T-antigen has been inserted. The 293 T cells were cultured in DMEM medium (GIBCO, catalog number 12800017) supplemented with 1.5 g/L NaHCO_3_. The culture medium consisted of 90% DMEM and 10% high-quality fetal bovine serum. The cells were maintained at a temperature of 37 degrees Celsius and a carbon dioxide concentration of 5%.

#### Cell transfection

Transfection procedures adhered to the manufacturer’s instructions, utilizing Lipofectamine 2000 (Invitrogen) for 293 T cells and Lipofectamine 3000 (Invitrogen) for GC-2spd (ts) cells. All DNA plasmids were subsequently removed. The plasmid was then introduced into 293 cells, and positive cells were screened and expanded.

#### Male mouse neuron primary cells

Adult mouse fertilized egg cells were first selected as the cellular carriers. Subsequently, sgRNA expression vectors were constructed. The donor plasmid (provided by Gempharmatech Co., Ltd) was linearized using BbsI, and sgRNA-oligos were synthesized, annealed, and ligated into the BbsI site of the pUC57-sgRNA expression vectors containing the T7 promoter. The *Maoa* c.1409 T > C mutation mouse was created through *in vitro* transcription of sgRNA. Donor vectors were prepared, and a microinjection of Cas9, sgRNA, and donor material was performed on fertilized eggs from C57BL/6 J mice, facilitating homologous recombination and yielding F0 mice. Furthermore, F0 mice lacking off-target effects were bred with WT mice, giving rise to a new generation of F1 male mice. PCR and sequencing confirmed the stable transfer of the target mutation to the offspring. Female mice and male mice of appropriate age were reared in a cage, each male mouse was paired with three female mice, and the female mice were anesthetized on the 15th day after conception and subjected to cesarean section, and the midbrain region of fetal mice was prepared into a single-cell suspension and then seeded in cell culture plates. The seeding density is 1*10^^6^cell/ml, Male Mouse Neuron Primary Cells were cultured in the intact culture medium of the mouse dopamine neurons.

### Experiment grouping

Littermate mice were randomly assigned to groups every 5 cages. Adult mice weighing between 22 and 26 grams were selected through complete randomization before each experiment. All experimental procedures were conducted as single-blind experiments, meaning that the significance of the group assignments remained undisclosed to experimenters and data processors.

### Genomic DNA extraction and sequencing

The DNA samples underwent sequencing in the second generation at Shenzhen BGI Gene Technology Service Co., Ltd. To obtain rat tail samples, adult C57 mice aged 6–7 weeks were randomly selected. Approximately 0.5 cm of the mouse tail end was carefully excised, taking into consideration the age and thickness of the mouse to ensure uniform tail sample volumes. Each mouse tail segment was placed into a numbered 1.5 mL centrifuge tube, and the tube was securely closed. Rat tail DNA was then extracted utilizing the DNA Tissue Extraction Kit, with DNA concentration measurements employed to maintain the template concentration at 100 ng/μL. Specific primers were designed for PCR amplification of the *Maoa* gene, and the resulting PCR products underwent identification. Subsequently, the nucleobase sequence was determined through peak sequencing, enabling the mapping of the mouse *Maoa* gene.

### Western blot and MAOA ELISA kit

Mouse brain tissues or cells were treated with RIPA buffer (containing 1 M medta, 1 mM PMSF, and 10 mg/mL aprotinin) for 30 min. Subsequently, the samples were centrifuged at 1000 rpm for 5 min. Mitochondria were isolated using a mitochondrial extraction kit, and the resulting pellet was resuspended and mixed with a loading buffer. Protein denaturation was achieved by heating at 100°C for 5 min. Electrophoresis was performed on ice using a 10% precast gel, initially at 80 V for 15 min and then switched to 140 V for an additional 30 min. The separated proteins were subsequently transferred using a membrane transfer apparatus. The membrane was then cut into strips, and primary antibodies, including MAOA (1:500, Mouse, CST:#75330) and GAPDH (1,1,000, Mouse, CST:# 5174S), were applied for incubation. Following incubation, the bands were visualized using a scanner. Quantification of the immune response bands was conducted across 3 ~ 5 independent experiments using ImageJ software. Results were normalized with the corresponding actin or HA-RFP bands serving as loading controls. The MAOA protein content in the mouse ventral tegmental area (VTA) was determined using an ELISA kit (Shanghai Jiang Lai Biotechnology Co., Ltd., China), following the manufacturer’s protocols. MAOA activity was assessed using the MAO-Glo™ Assay Kit (Promega (Beijing) Biotech Co., Ltd).

### Quantitative PCR and semi-quantitative PCR

VTA brain regions from cultured cells or mouse brains were isolated and subjected to RNA extraction using an RNA extraction kit (Accurate Biology). Subsequently, reverse transcription was carried out to convert RNA into DNA utilizing a reverse transcription kit (Accurate Biology). Quantitative PCR (QPCR) was performed using the LightCycler96 instrument. Quantitative normalization was achieved using endogenous GAPDH as a reference gene. The primer pairs used for QPCR are detailed in the Table 2. Prior to QPCR, primer amplification efficiency was assessed, and the optimal number of cycles was determined in advance to ensure that the amplification of each primer remained within the exponential range (Chen et al., 2023). Data analysis was performed by the formula: 2^-ΔCt^(ΔCt = Ct*_maoa_* – Ct_GAPDH_).

**Table 2:** Primers used for QPCR.

Primers	Sequence (5′ → 3′)
Mus-Gabra5-108F	TCCACAACGGCAAGAAGTCC
Mus-Gabra5-108R	CAGAGATTGTCAGACGCATGG
Mus-Gabra2-177F	GGACCCAGTCAGGTTGGTG
Mus-Gabra2-177R	GGGCCAAAACTGGTCACGTA
Mus-Gabra6-88F	GCTGATTGCCCCATGAGATTG
Mus-Gabra6-88R	CAGTTTTAGGATAAGCATAGCTCCC
Mus-Drd1-108F	AGGTTGAGCAGGACATACGC
Mus-Drd1-108R	TTGCTTCTGGGCAATCCTGT
Mus-Dbh-106F	CTGGGGTCCTGTTTGGAATGT
Mus-Dbh-106R	TCACTCCAGGCATCCGCAA
mus-*Maoa*-F	TATGTGAGGCAGTGTGGAGGTA
mus-*Maoa*-R	CCAAGGAGGACCATTATCTGTTCA
GAPDH-F	TGTGTCCGTCGTGGATCTGA
GAPDH-R	TTGCTGTTGAAGTCGCCACGAG

### mRNA and protein half-life assays

Fetal mouse neuron primary cells were seeded in a 24-well plate and allowed to incubate for 12 h. Subsequently, they were rinsed with PBS and the culture medium was replaced. After 72 h of culture, cells were washed three times with PBS and then supplied with serum-free medium, with 1 mL added to each well. The study design included setting up three parallel groups for both the blank control and the experimental groups. In the experimental group, 10 μL of Actinomycin D (ActD) and cycloheximide (CHX) were added to each well (ActD: 25 μg/mL, CHX: 40 μg/mL) in serum-free medium to disrupt mRNA transcription and protein biosynthesis. Cells were subsequently collected at 0, 4, 8, 12, and 24 h, with total RNA and protein extraction performed. Total RNA was utilized to assess mRNA stability, while the extracted proteins were employed for monitoring protein stability.

### Di luciferase assay

Luciferase reporter assay is a reporter system that uses fluorescein as a substrate to detect the activity of firefly luciferase (F-Luc). Taking advantage of the chemiluminescence reaction of luciferase binding to substrates, the transcriptional regulatory elements of the gene of interest can be cloned upstream/downstream of the firefly luciferase gene to construct a luciferase reporter plasmid. Cells are then transfected, lysed after appropriate stimulation or treatment, and luciferase activity is measured. The effect of different stimuli on the regulatory element of interest before and after stimulation or by the level of luciferase activity can be determined. In the psiCheck2 plasmid, the GAT codon in Rluc is replaced with the GAC codon to represent two different SNP genotypes. The binding efficiency to luciferase was assessed by comparing the fluorescence signal intensities of two different codons, and the experimental results were used to characterize the binding efficiency of GAC and GAT codons. Subsequently, 293 T cells were transfected with both the wild-type and mutant plasmids. The purpose was to compare the firefly luciferase signal and the Renilla luciferase (Rluc) signal, with Rluc serving as the mutation target and firefly luciferase as the internal reference normalization signal. Both plasmids were co-transfected into 293 T cells. After 48 h, cell lysate (1 mL) was added, followed by a 5-min incubation at room temperature to ensure complete cell lysis. The lysate was then centrifuged at 10,000 g for 5 min, and the resulting supernatant was collected for testing. The firefly luciferase assay and Renilla luciferase assay buffers were thawed and brought to room temperature. The Renilla luciferase test substrate (100×) was placed on ice for later use. The chemiluminescence instrument was turned on following the manufacturer’s instructions. The 100 μL of lysate supernatant was added to a 96-well luminescent plate, and 100 μL of firefly luciferase detection working solution was added for the assay. The contents were mixed thoroughly, and luminescence readings were obtained from the instrument, with an integration time of 5 s. Subsequently, 100 μL of Renilla luciferase detection working solution was added, and the contents were mixed. Luminescence values were measured on the instrument, again with an integration time of 5 s. To determine the degree of activation of reporter genes in different samples, the RLU (Relative Light Units) value obtained from the Renilla luciferase assay was divided by the RLU value obtained from the firefly luciferase assay when using firefly luciferase as the internal reference.

### RNA pull-down

After collecting the primary cells, transfer them into EP tubes, and centrifuge at 3000 rpm for 3 min. Discard the supernatant and add 1 mL of protein lysis buffer containing PMSF to the cell pellet. Shake well and place the mixture on ice, allowing it to lyse for 30 min. Then, centrifuge the lysate at 12,000 rpm for 5 min at 4°C to collect the supernatant. Take 50 μL of magnetic beads and wash them with 1 mL of 1x TBS, placing them on a magnetic rack to remove the supernatant. Repeat this washing process with magnetic beads three times. Then, add 1 mL of 1x TBS, mix well, and divide the magnetic beads into two parts, labeled as WT and MUT, with 500 μL in each portion. Place them on a magnetic rack to remove the supernatant. The probe sequences used are as follows: rs1137070-WT: GCCAAGAAGGATATATGTT and rs1137070-MT: GCCAAGAAGGACATATGGGTT (T for wildtype and C for mutant sequence). Simultaneously, magnetic beads with the wildtype probe were used as a control group. Wash the magnetic beads twice with nucleic acid incubation buffer, followed by two washes with protein incubation buffer. Place the magnetic frame on the magnetic separation to collect the supernatant. The protein lysate is diluted with protein incubation buffer and added to the RNA-magnetic bead complexes. The mixture is incubated at room temperature for 2 h to form protein-RNA probe-magnetic bead complexes. Afterward, remove the supernatant, wash the magnetic beads 6–7 times with protein incubation buffer, and collect the pellet. Finally, add 60 μL of PBS and 15 μL of 5x Loading Buffer to the magnetic beads, and incubate in a water bath at 95°C for 5 min. Perform SDS-PAGE gel electrophoresis using a 5% gel concentration for the separating gel. Set the voltage to 90 V for the upper gel and electrophorese for approximately 30 min (ensure the marker enters the separating gel). Adjust the voltage to 130 V for the lower gel and electrophorese for about 90 min. Subsequently, stain the gel with Coomassie Brilliant Blue for quality assessment. Send the gel for mass spectrometry identification and perform bioinformatics analysis to identify unique and common proteins between the wildtype and mutant probes.

### Mass spectrometry chromatographic conditions

Mobile phase solutions were prepared as follows: Mobile Phase A consisted of 100% water with 0.1% formic acid, while Mobile Phase B was composed of 80% acetonitrile with 0.1% formic acid. To dissolve the lyophilized powder, 10 μL of Mobile Phase A solution was used. The solution was then centrifuged at 14,000 g at 4°C for 20 min, and 1 μg of the resulting supernatant was injected for liquid detection. Liquid chromatography elution conditions are detailed in Table 1. The analysis was performed using a Q Exactive HF-X mass spectrometer equipped with a Nanospray Flex™ (NSI) ion source. The ion spray voltage was set to 2.1 kV, the ion transport tube temperature was maintained at 320°C, and mass spectrometry was conducted in data-dependent acquisition mode. The full scanning range for mass spectrometry was set to m/z 350–1,500, with the primary mass spectral resolution set at 120,000 (at 200 m/z). The AGC (Automatic Gain Control) was set to 3 × 10^^6^, and the maximum injection time for the C-trap was 80 ms. In the full scan, the top 40 ion signals with the highest intensity were selected for fragmentation using the high-energy collision cleavage (HCD) method for secondary mass spectrometry detection. The secondary mass spectrometry resolution was set to 15,000 (at 200 m/z), with an AGC of 5 × 10^^4^ and a maximum injection time of 45 ms. A collision energy of 27% was applied to generate peptide fragment spectra, resulting in the acquisition of raw data for mass spectrometric analysis (.raw).

### Bioinformatics and statistical analysis

The gene database from NCBI (https://www.ncbi.nlm.nih.gov/snp) and the genome database from Ensemble (http://grch37.ensembl.org/index.html) were utilized to access relevant data. Snapgene software was employed to perform sequence homology comparisons between the regions encoding the *Maoa* gene in humans and mice. Additionally, an analysis of the frequency of this synonymous mutation in different ethnic groups was conducted using data from the human 1,000 Genomes database (https://ftp.ncbi.nlm.nih.gov/1000genomes). To predict potential mRNA secondary structures, the Mfold web server (http://www.unafold.org/) was employed to determine mRNA folding forms and minimum free energy. The design of primers for this study was facilitated by primer Premier Software version 5.0 (Premier Biosoft International) and the synthesized primers were provided by Hunan Accurate Biology Bioscience. The abscissa of the KEGG pathway diagram is the ratio, the ordinate is the individual KEGG pathway entries, the color represents the enrichment degree [−log10 (*P* value)], and the circle size represents the number of genes. For statistical analysis, GraphPad Prism 8.0.2 (GraphPad Software, La Jolla, CA, United States) was used. The sample size (n) typically represents the number of experimental replicates, as indicated in the legend. In experiments involving mouse brain tissue, the sample size (n) specifies the number of mice used. Data obtained from experiments were presented as mean ± SD. The normal distribution of data was assessed using QQ plots in GraphPad Prism 9.0.2, along with an F-test to examine the equality of variances. A *p*-value >0.05 in the F-test indicated homogeneity of variance. All statistical tests were two-tailed, and statistical significance was defined as *p* < 0.05 (*p < 0.05, **p < 0.01, ***p < 0.001).

## Results

### The sequencing results of the mouse model with *Maoa* gene mutation were stable and had high homology with human genes

**Figure 1:**
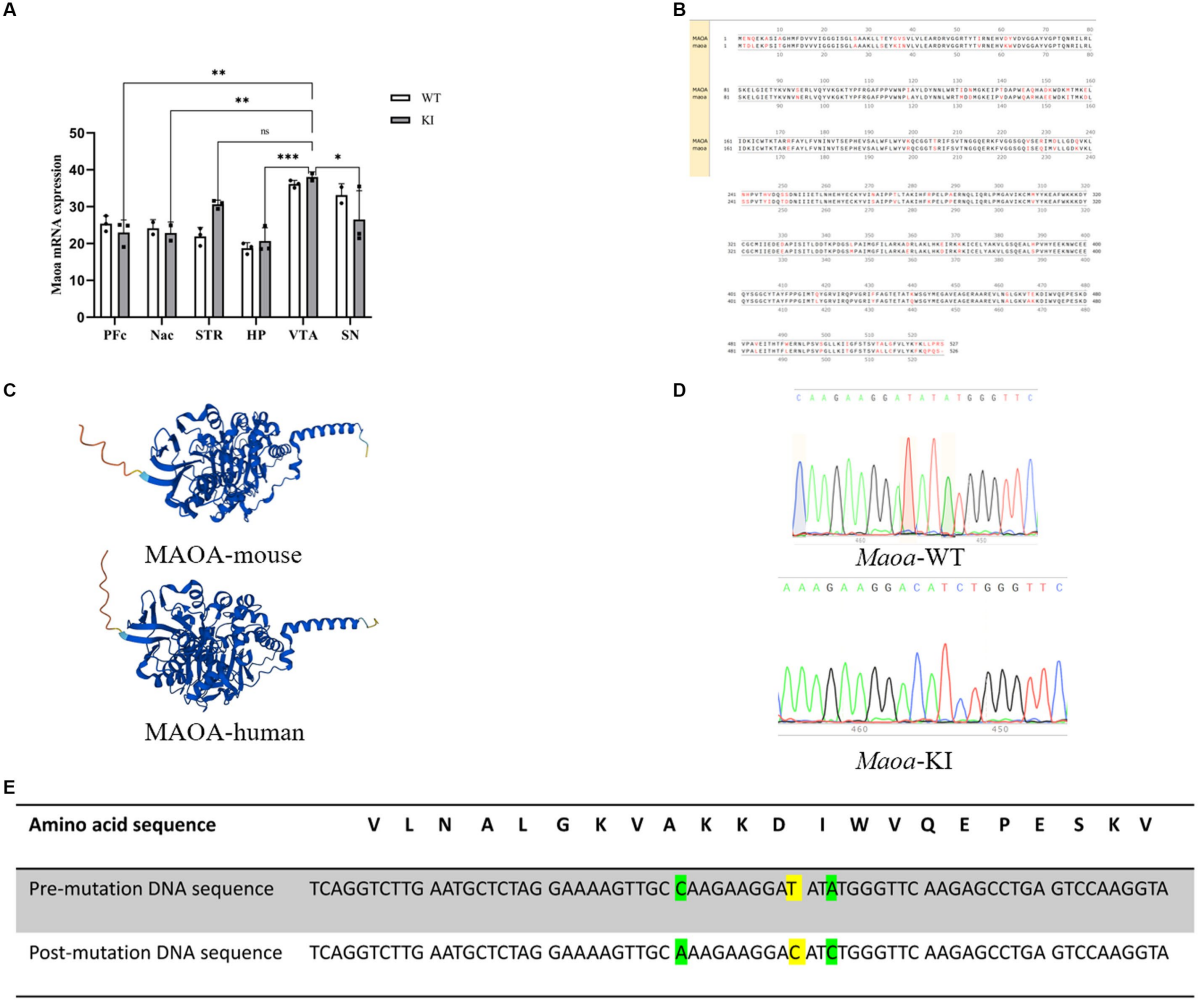
Species homology comparison results and gene use frequency analysis of species at gene mutation sites **(A)** *Maoa* c.1409 T > C in different ancestry before and after gene frequency. **(B)** Schematic diagram of the homology of the amino acid sequence in the *Maoa* gene, the total length of the *Maoa* amino acid sequence is 526aa, the same length as the human amino acid sequence is 460aa, so the homology is 87.4525%. **(C)** Prediction of the alpha fold of *Maoa* protein structure in humans and mice. RMSD is 0.65 and TM-score is 0.98, Target coverage is 98%.Template modeling score (TM-score) is a measure of topological similarity between the template and model structures, and it is 0.98 indicating high similarity. **(D)** Sequence plots of wild-type (WT) mice and knock-in (KI) mice *Maoa* gene sequencing. The yellow marker represents the mutation site, and the codon of the 470th aspartate amino acid is mutated from GAT to GAC. **(E)** Mutation sites and corresponding amino acid sequences and DNA sequences control diagram, yellow indicates rs1137070, c.1409 T > C synonymous mutations, green indicates rs76204226, c.1401C > A and rs766439090 c.1413A > C synonymous mutations.

Based on the information gathered from GWAS and the RefSeq Genomic Database, we explored the gene frequencies of the *Maoa* c.1409 T > C variant across different ethnic groups. According to the 1,000 Genomes database, the allele frequency of this SNP is 57.4% in the Asian population, 29.4% in the European population, and 37.24% in the African population (Figure 1A). To assess whether mice could serve as suitable models for synonymous mutations at this site, we examined the protein and nucleotide sequence similarities between the human and mouse *Maoa* genes. Comparing the homology of exons within the *Maoa* gene revealed an identity of 87.4525% between humans and mice (Figure 1B). Additionally, we used AlphaFold (https://www.alphafold.ebi.ac.uk/) to predict the highly reliable computational structure of the MAOA protein. The MAOA computational structure model from AlphaFold was subjected to Pairwise Structure Alignment in the SCB PDB database, demonstrating a good alignment (Figure 1C). Mice were selected as animal models, and a mouse model carrying the c.1409 T > C mutation was generated using CRISPR/Cas9 technology. After breeding, DNA was extracted from the tails of wild-type and KI (knock-in) mice, and gene sequencing was performed (Figure 1D). Sequencing results indicated that, among the four mutation sites tested (rs76204226, c.1401 C > A, rs1137070, c.1409 T > C, rs766439090, c.1413 A>), only the c.1409 T > C mutation was present in the *Maoa*_Exon14 region. Importantly, there were no mutations at other sites, and no evidence of non-homologous recombinant repair (NHEJ) or hetero peaks were observed in the sequencing data. The amino acid sequence analysis confirmed that both human and murine *Maoa* genes encoded aspartic acid at amino acid position 470 (Figure 1E). It is worth noting that codon usage preferences for this synonymous mutation can vary significantly among different racial populations. A comprehensive study using large-scale whole-genome sequencing data of the Chinese population [the China Metabolic Analytics Project (China MAP)] reported an allele frequency of 70% for this gene (Cao et al., 2020).

### *Maoa* c.1409 T > C synonymous mutation affects MAOA expression and activity

**Figure 2:**
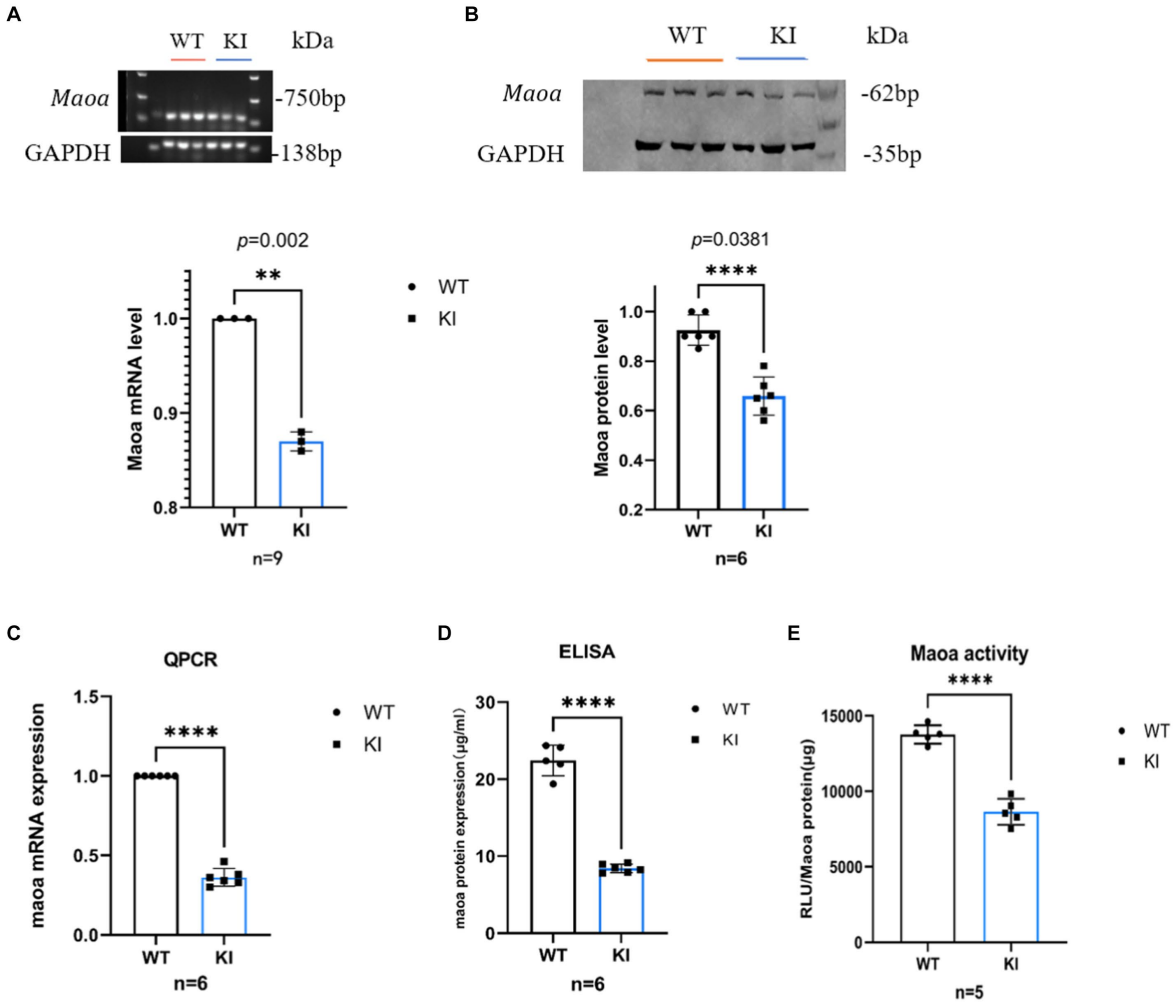
Synonymous mutations reduce *Maoa* transcription and translation levels in mouse brain regions. **(A)** mRNA expression of the *Maoa* gene in the brain regions of wild-type mice and KI mice (*n* = 3, ****p < 0.0001).**(B)** Mutant *Maoa* (KI) mice reduced the transcription level of the *Maoa* gene (*n* = 5, ** *p* < 0.01). **(C)** Western Blot detected the expression of MAOA protein in the (ventral tegmental area, VTA) brain region of mice (*n* = 3, **p < 0.01). **(D)** ELISA enzyme-linked kit detected MAOA expression, and the level of MAOAin the brain region of mice in the KI group was significantly lower than that in the WT group (*n* = 6, **p < 0.01). **(E)** The MAOA enzyme activation kit showed that the MAOA enzyme activity of KI mice was significantly lower than that of KI mice (*n* = 5, ****p < 0.0001).

To assess the impact and magnitude of the synonymous mutation *Maoa* c.1409 T > C on the *Maoa* gene product at the animal level, *Maoa* gene expression was evaluated in adult WT and KI mice following brain extraction. In male mice, mRNA expression in 8-week-old *Maoa*-mutant mice was significantly lower than in WT mice (Figures 2A,B, *p* < 0.0001). Following the observed decrease in mRNA expression, changes in *Maoa* protein levels were further examined using western blot (WB) and enzyme-linked immunosorbent assay (ELISA). The results demonstrated that *Maoa* expression in the brains of 8-week-old KI male mice was significantly lower than that in WT mice (Figures 2C,D, *p* < 0.01). Consequently, *Maoa* c.1409 T > C exerts an influence on both transcription and translation, aligning with previous findings regarding synonymous mutations in the literature. Additionally, differences in the enzyme activity of *Maoa* expression products were measured, with enzyme activity found to be lower in KI mice compared to WT mice (*p* < 0.0001, Figure 2E).

### Effects of synonymous mutations of rs1137070 on MAOA protein and mRNA stability

**Figure 3:**
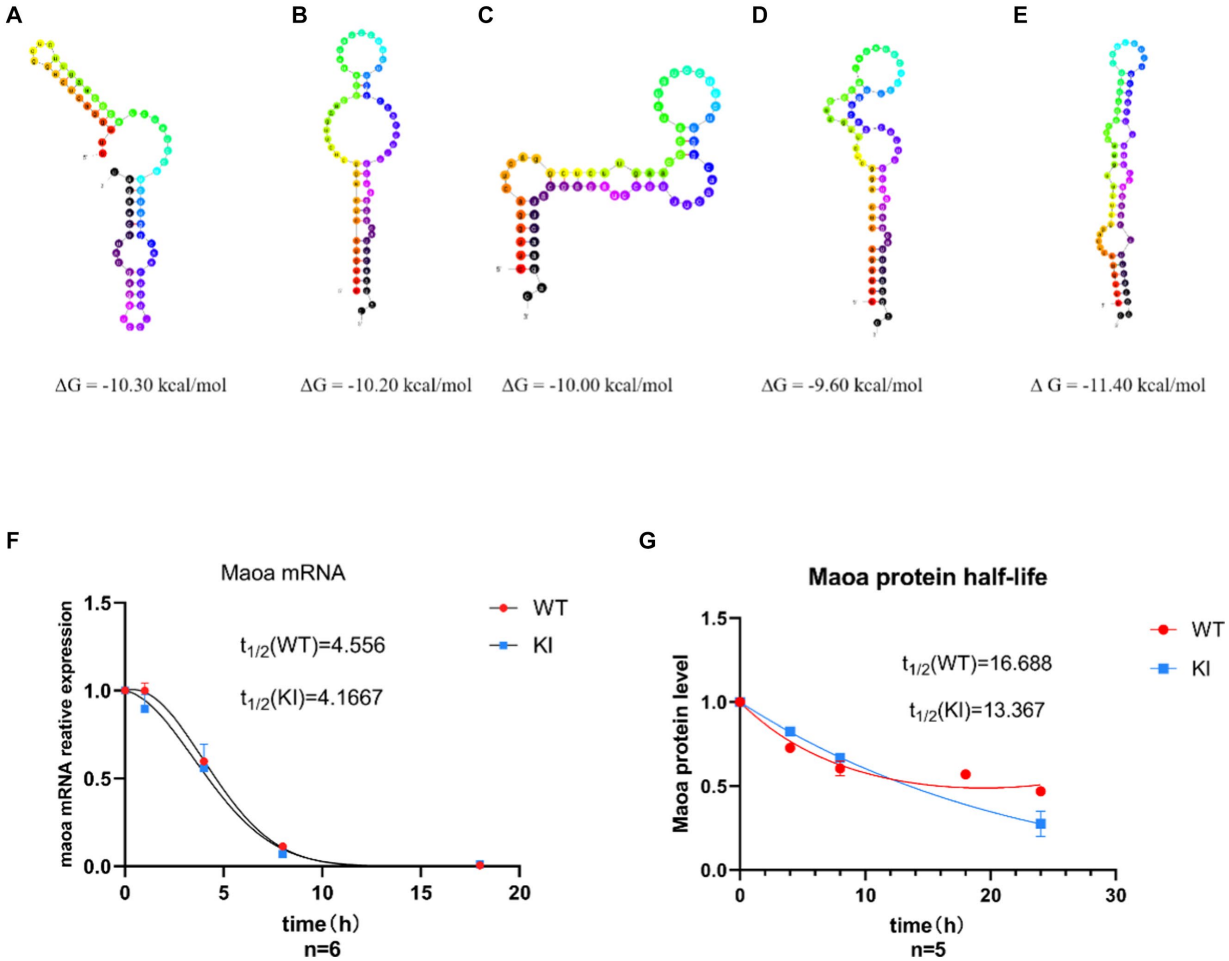
Synonymous mutations affect *Maoa* mRNA secondary structure and protein stability. **(A–D)** *Maoa* mRNA secondary structure prediction results in WT mice **(E)** *Maoa* mRNA secondary structure prediction results in KI mice **(F)** QPCR detected the effect of different codons on the half-life of *Maoa* mRNA, and the half-life of the KI group was smaller than that of the WT group. **(G)** WB detected the effect of different codons on protein stability, and the stability of group K I protein was poor (*n* = 6) and the difference was statistically significant.

Differences in synonymous codons can often lead to alterations in mRNA secondary structure and stability. To further investigate whether synonymous mutations impact the structure and stability of mRNA and proteins, we examined mRNA half-lives. Initially, we utilized the UNA Fold Web Server (http://www.unafold.org) to predict the secondary structure and minimum free energy of mRNA. The results indicated that the synonymous mutant mRNA sequences caused changes in mRNA secondary structure and minimum free energy. Specifically, in *Maoa*-KI mice (Figures 3A–D), these values decreased by 1.10 kcal, 1.20 kcal, 1.40 kcal, and 1.80 kcal, respectively, compared to *Maoa*-WT (Figure 3E). Therefore, the predictions suggest that the mRNA structure in WT mice is more stable. Subsequently, to further assess differences in mRNA half-life, we used Actinomycin D (Act D) to block *Maoa* gene transcription. After RNA extraction from mouse neuronal primary cells, the amount of RNA product was detected by QPCR. The results demonstrated that mRNA half-lives were longer and more stable in the WT group compared to the KI group (Figure 3F). To further verify the impact of synonymous codons on protein stability, proteins were extracted after adding Actinomycin (CHX) to the neuronal primary cell culture medium for Western Blot experiments. The findings indicated that the WT group exhibited greater stability than the KI group (Figure 3G).

### Detection of dominate codon by Di luciferase

**Figure 4:**
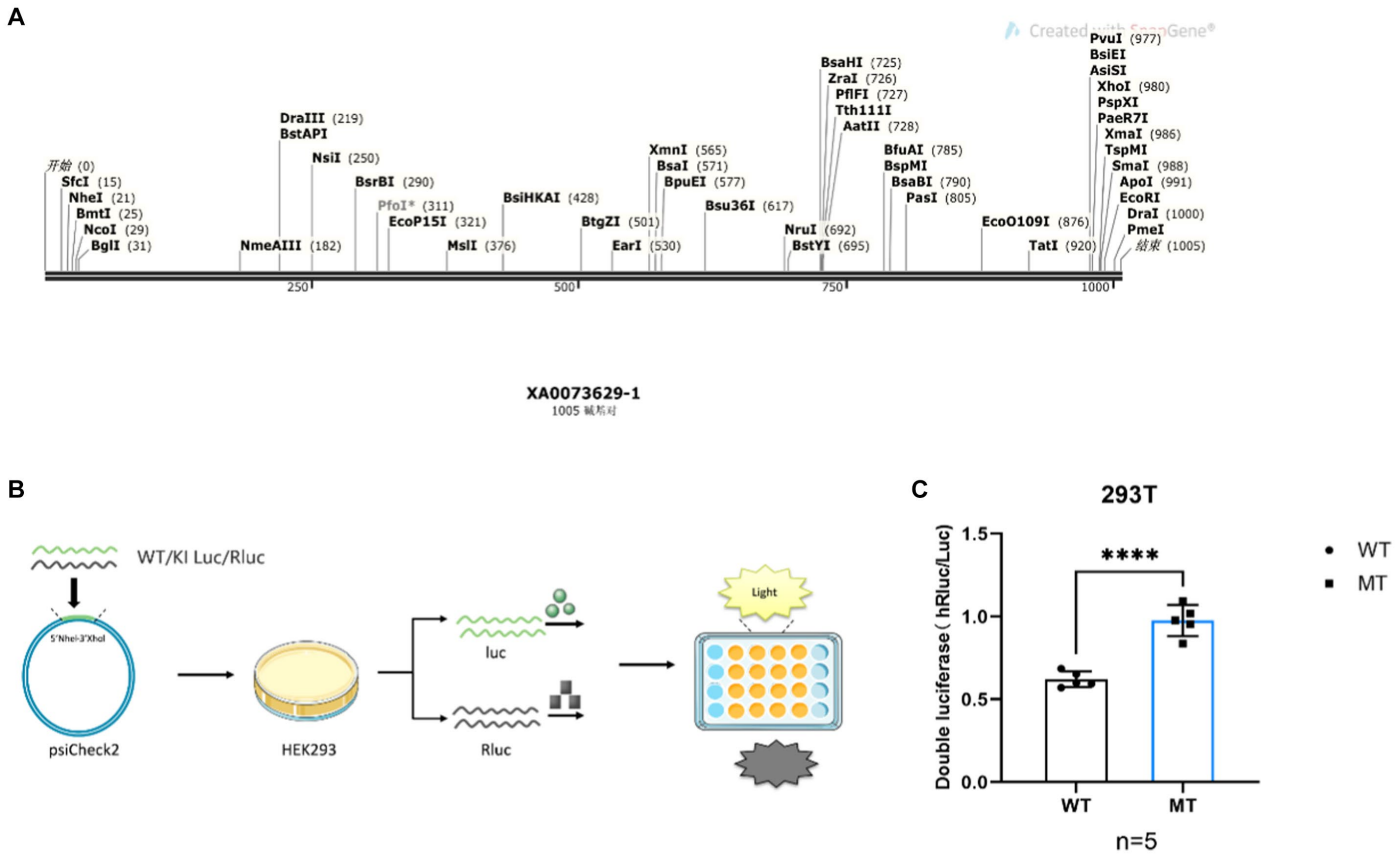
*Maoa* point mutation sequence luciferase activity flash strong is the dominant codon. **(A)** DNA sequences carrying different codons Contains 6 GAC/GAT codons, respectively. **(B)** Schematic diagram of the diluciferase experiment. **(C)** Dual-luciferase reports the luciferase activity intensity (*p* < 0.0001) of both gene sequences.

To investigate potential preference differences in transcription and translation of codons (Gu et al., 2013), we designed DNA sequences with varying GAC and GAT content (Figure 4A). These sequences were inserted into the psiCheck2 plasmid (Figure 4B) and co-transfected into 293 T cells for the assessment of codon binding efficiency through dual-luciferase experiments. By comparing firefly luciferase signaling in wild-type and mutant plasmids with sea kidney luciferase signaling, it was evident that the luciferase activity of the mutated GAC codon reporting plasmid was significantly enhanced compared to the GAT codon (Figure 4C, ****p < 0.0001). Consequently, when compared to the dominant GAT codon, the GAC codon exhibited increased mRNA secondary structure stability (Figures 3A–E).

### RNA pull-down experiments screen translational regulatory proteins

**Figure 5:**
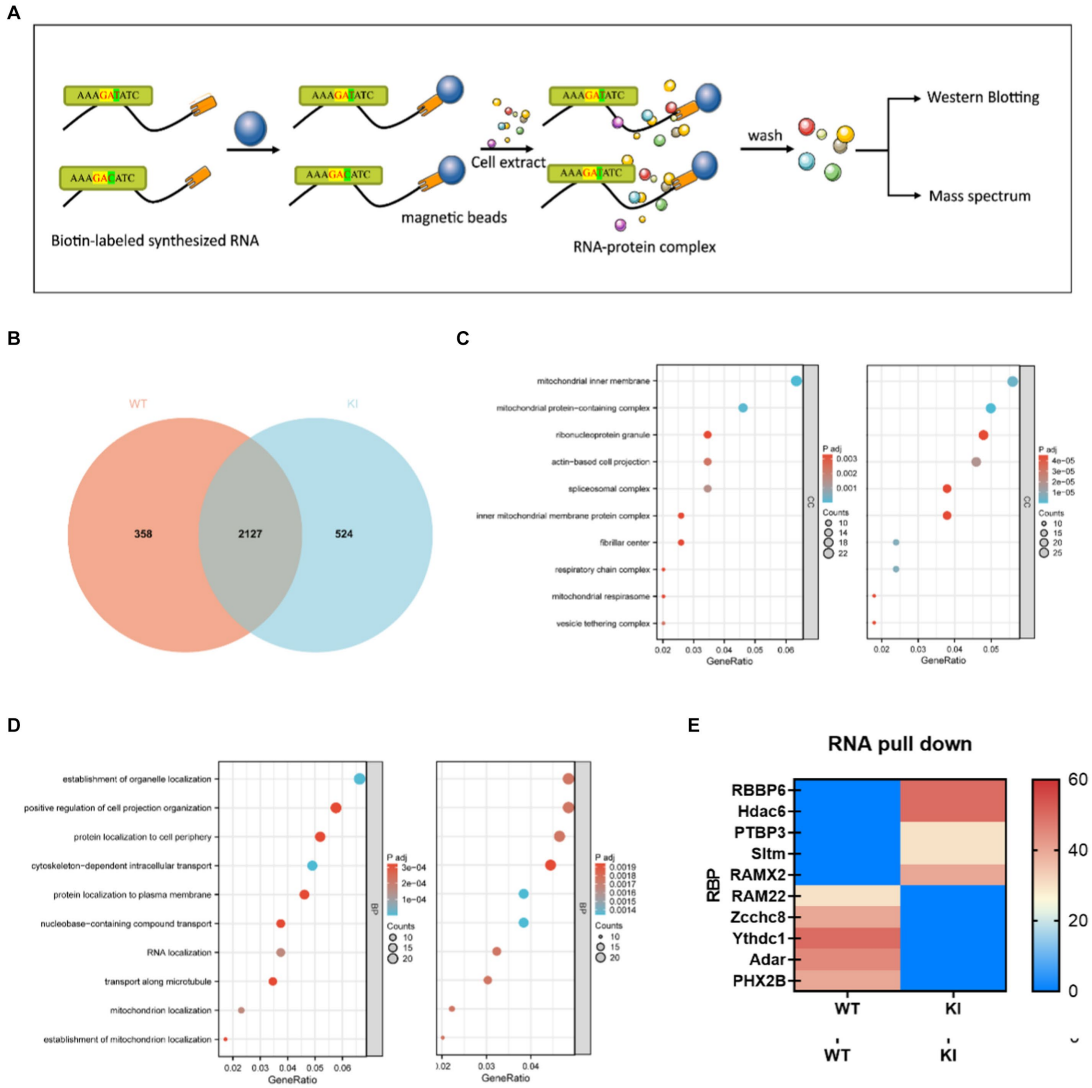
Synonymous mutations affect *Maoa* mRNA secondary structure and protein translation initiation. **(A)** Schematic of RNA pull-down experiment. Biotin-labeled RNA probes are perfused with primary cell extracts, and this complex binds to streptavidin-labeled magnetic beads and thus separates them from other components in the incubation solution. Elute proteins on complexes for mass spectrometry or western blot detection. **(B)** Differential protein Veen diagram **(C,D)** Differential protein GO analysis **(E)** Liquid chromatography–tandem mass spectrometry (LC–MS/MS) screening of differential protein with wild type (WT) and mutant (Mut) synthetic RNA.

Several studies have suggested that the binding of RNA-binding proteins (RBPs) can influence the secondary structure stability of mRNA, thereby impacting the accuracy and proper execution of splicing. To further validate differences in binding between wild-type and mutant-type sequences, we conducted RNA pull-down experiments to assess the affinity variations between these sequences and RNA-binding proteins. Short RNA sequences were directly synthesized and biotin-labeled at the N-terminus (refer to Materials and Methods for details). RNA-binding proteins recognizing WT (ACCGAGAAAGATATCTGGGT) and KI (ACCGAGAAAGAATCTGGGT) probes in mouse primary cells were enriched through RNA pull-down experiments, and the identified RBP proteins were analyzed using liquid chromatography–tandem mass spectrometry (LC–MS/MS). Among the 2,651 proteins associated with the KI sequence and the 2,485 proteins associated with the WT sequence, there were 524 KI-specific proteins and 358 WT-specific proteins (Figure 5A). GO analysis of sequence-specific RBP proteins for KI and WT sequences (Figures 5B,C) revealed the top ten specifically binding proteins based on enrichment degree. Notably, RBBP6, Hdac6, PTBP3, RAMx2, and Sltm exhibited high-affinity binding to KI sequences and were linked to translation regulation, with wild-type sequences largely hindering this binding affinity. In contrast, WT sequences showed a higher specificity for RAM22, Zcchc8, Ythdc1, and Adar PHX2B (Figure 5E).

### RNA-seq transcriptome sequencing analysis

**Figure 6:**
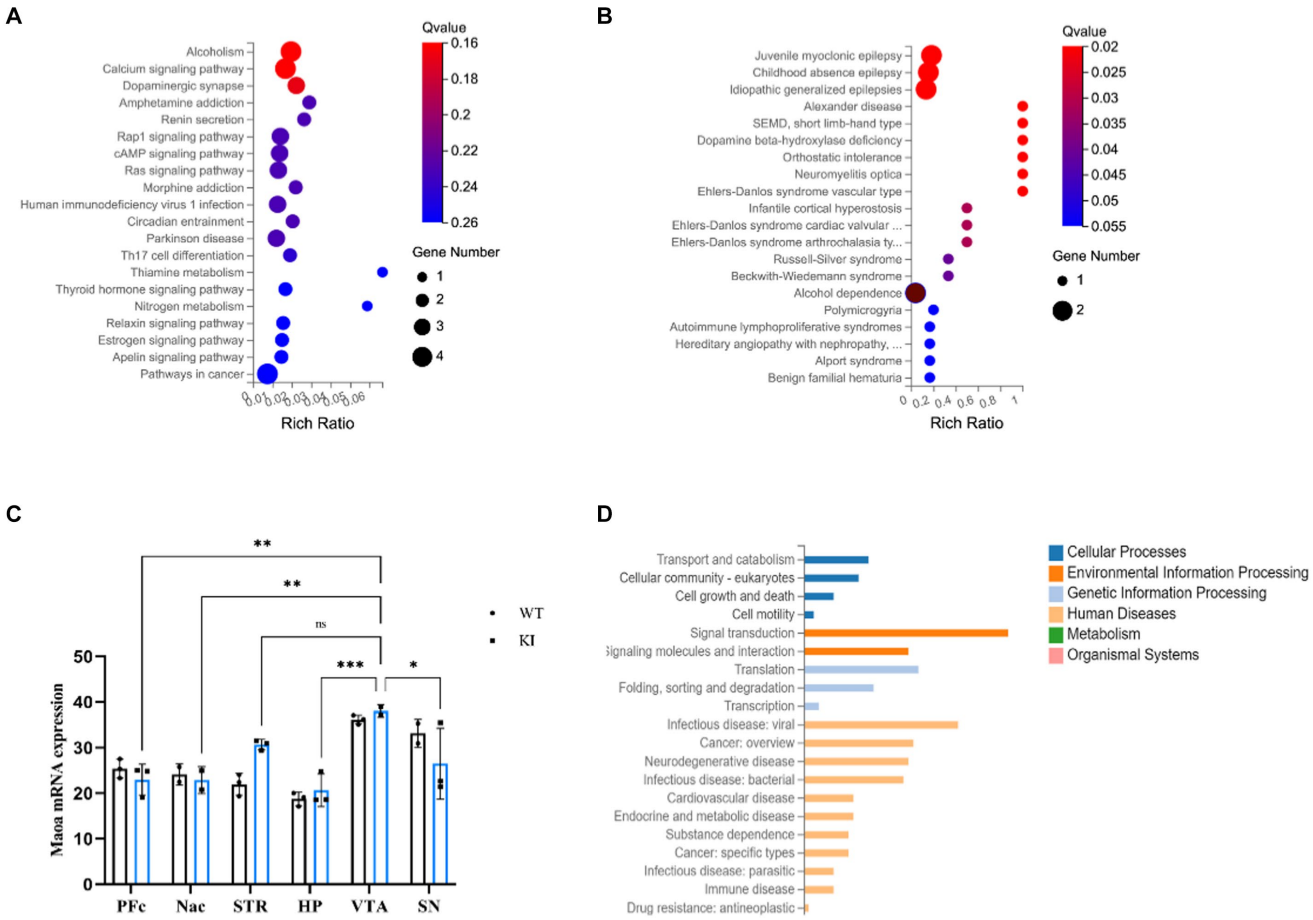
**(A)** Differential gene KEGG enrichment bubble chart, A is the pathway enrichment map, **(B)** is the metabolic enrichment map. **(C)** The expression of the *Maoa* gene in six brain regions. **(D)** KEGG pathway classify of PFc.

To investigate whether this synonymous mutation also influences gene expression and the differential expression of associated genes. The RNA-seq analysis focused on six brain regions associated with the reward system, with special attention to the VTA as a key hub in reward and motivation processing within the midbrain limbic dopaminergic system. The results of RNA-seq revealed that the *Maoa* gene exhibited the highest transcriptional activity in the VTA brain region (Figure 6C) (*p* < 0.01). First, we counted the number of differential genes in different brain regions, and the results showed that the number of differential genes in VTA and Pfc brain regions was relatively large, so the differential genes of these two brains were selected for further analysis. Firstly, KEGG analysis of the differentially expressed genes in the brain region of VTA was compared between KI and WT, and the results showed that 6 differentially expressed genes were screened (Figures 6A,B). Notably, we found that DNA-binding transcription factor activity, sequence-specific DNA binding, and RNA polymerase II proximal promoter sequence were linked to the mutation (Figure 6D). This outcome suggests that synonymous mutations can indeed impact the up-and down-regulation of expression in other genes.

## Discussion

*Maoa* plays a pivotal role in various metabolic processes involving monoamines and holds significant importance in the metabolism of neuroactive and vasoactive amines across both the central nervous system and peripheral tissues. Moreover, *Maoa* serves as a crucial candidate for genetic investigations into various mental disorders (Ziegler and Domschke, 2018). The primary focus of this study is to delve into the mechanisms underlying the influence of *Maoa* gene polymorphisms and *Maoa* expression in individuals with T and C genotypes who are addicted to smoking, thereby shedding light on the role in addiction.

Based on GWAS and China Map data, it has been observed that different human ancestry exhibit two genotypes, C and T, for this gene, with varying frequencies (Cao et al., 2020). These genetic differences have been correlated with disparities in brain function (Shen et al., 2019). Further analysis of different populations found that the rs1137070 SNP was significantly associated with smoking susceptibility, heroin addiction, hostility and depression, Parkinson’s disease and autism, bipolar disorder, schizophrenia, and other disorders. Genome-wide analysis of the population also found that this locus was significantly associated with psychiatric disorders in Europeans (*p* = 0.00014, T allele OR = 1.466) and Asians (*p* = 0.015, OR = 1.148 for T alleles). By comparing amino acid sequences and protein structures, it has been discovered that *Maoa* in humans and mice share a high degree of similarity, with an impressive 87% homology (Figure 1B) and an exceptional 98% structural similarity (Figure 1C). Consequently, a mouse model featuring a synonymous mutant *Maoa* gene with a C base was developed using CRISPR-Cas9 technology, effectively simulating two gene polymorphisms observed in humans. In this research, the primary focus has been on investigating the *Maoa* gene from the perspective of gene expression regulation.

It’s noteworthy that SNPs are synonymous mutations, typically assumed not to influence gene expression. However, there is a growing body of evidence indicating that synonymous codons play a crucial role in determining gene expression levels and protein structure (Liu et al., 2021b). Synonymous mutations primarily impact gene expression by influencing transcriptional elongation speed, thereby regulating translation efficiency and accuracy. Hence, in the examination of *Maoa* gene expression, the study began by measuring the changes in MAOA levels and exploring whether the GAC codon in this sequence resulted in higher gene expression compared to the *Maoa*-WT group (Figures 2A–D). The findings demonstrated that both mRNA and protein levels of the *Maoa* gene were relatively lower in the CC genotype group compared to the WT group at 6 weeks of age. Subsequently, *Maoa* enzyme activity was assessed, revealing that the CC genotype displayed lower MAOA enzyme activity (Figure 2E), consistent with previous findings (Agatsuma et al., 2006). It’s worth noting that synonymous mutations can alter protein folding due to differences in codon translation speeds (Yu et al., 2015), potentially leading to changes in enzyme activity. Given that dopamine metabolism in rodents is primarily influenced by *Maoa* (Paterson et al., 1991), a decrease in MAOA protein levels and reduced enzyme activity can result in abnormal levels of dopamine in the brain. This mutation primarily affects *Maoa* epigenetically.

Further molecular biology experiments are needed to conclusively determine whether synonymous mutations affect MAOA expression through codon usage. Different organisms exhibit preferences for specific codons, with mammals often favoring C/G in the wobble position (Hershberg and Petrov, 2008). Therefore, we hypothesize that GAC is a more favorable codon compared to GAT. According to the codon usage bias theory, codon preference can influence gene expression, sometimes leading to increased protein production (Zhou et al., 2016) or, in other cases, decreasing it (Yang et al., 2019). Our Western blot (WB) and quantitative polymerase chain reaction (QPCR) results indicated that the GAC codon led to a downregulation of protein expression (Figures 2A–D). Therefore, in the best codon theory, we believe that GAT is the dominant codon compared to GAC, and GAT has a higher amount of gene expression products than GAC.

Translation dynamics are intricately linked with mRNA structure (Liu et al., 2021b) and protein structure (Zhou et al., 2015), and different gene sequences can impact mRNA secondary structure (Gu et al., 2013). Therefore, we initially explored whether GAC is a dominant codon, thereby affecting mRNA secondary structure and protein structural stability. We investigated the effects of synonymous codons on mRNA secondary structure, mRNA half-life, and protein half-life. The prediction of mRNA secondary structure using UNFold revealed that the KI group sequence had a higher number of pairable bases and formed smaller loops. Additionally, it had a lower Gibbs free energy compared to the four possible secondary structures of the WT sequence, theoretically rendering it more stable (Figures 3A–E). Some research has indicated that dominant codons are enriched in stable mRNA sequences, while rare codons are associated with the formation of mRNA with shorter half-lives. The prediction results of mRNA secondary structure align with this theory.

However, QPCR experiments revealed that while the Maoa-KI secondary structure was stable, its half-life was not longer. The half-life of the KI group (4.1667 h) was actually shorter than that of the WT group (4.556 h) (Figure 3F). This discrepancy in half-life suggests that mRNA structure stability may be constrained by other factors. Subsequent RNA-pull down experiments further illustrated that the regulation of RNA-binding protein (RBP) could influence mRNA structural stability (Figure 4E). Following this, WB experiments were conducted to measure protein half-life. The half-life of the WT group was 16.668 h, whereas the half-life of the KI group was 13.367 h, indicating that the protein stability of *Maoa*-KI was inferior (Figure 3G). This finding aligned with the results obtained from the MAOA protein amount ELISA detection (Figures 2C,D).

Dominant codons significantly affect translation efficiency, as they exhibit higher binding efficiency to ribosomes and tRNA, leading to faster translation. Translation speed also impacts the folding rate of translation, which, in turn, affects protein function and structural stability (Zhao et al., 2017). To explore whether GAC is indeed a dominant codon compared to GAT, a DNA sequence containing six GAC/GAT sites was selected to expand the binding difference. The results of the dual-luciferase experiment revealed that GAC produced a higher sea kidney luciferase fluorescence signal (Figure 4C), confirming that GAC is the dominant codon. While all codons influence translation rate, some codons can be rapidly decoded and exhibit high-efficiency binding to ribosomes, whereas others have lower ribosome binding efficiency due to lower tRNA concentration (Bae and Coller, 2022). The findings indicate that more stable mRNA secondary structures are more challenging to unwind, resulting in lower translation efficiency (Kaushik et al., 2018).

The prediction of *Maoa*’s secondary structure suggests that it has a more stable structure, making it harder to unfold during the translation process. Consequently, translation efficiency is lower, and because its mRNA and protein half-lives are shorter and more susceptible to degradation, overall protein expression in KI decreases (Figures 2C,D). These observations highlight significant differences in maintaining mRNA and protein stability among different synonymous codons. Furthermore, it is important to consider that optimal codons do not necessarily lead to upregulated protein and mRNA levels, as these can also be influenced by mRNA stability and post-translational modifications. However, it is important to acknowledge that these results have certain limitations at the animal level. It is possible that mRNA cleavage and post-translational modifications by RNA-binding proteins (RBPs) may have contributed to the decrease in expression observed in the study.

The study of molecular mechanisms in gene expression regulation encompasses various processes, including transcriptional and post-transcriptional regulation, translation initiation, and post-translational modifications that collectively determine mRNA levels, protein structure, activity, and stability (Chu et al., 2014). Transcriptional regulation is generally influenced by the strength of the promoter region and upstream regulatory elements, while protein translation is often regulated by post-translational modifications and protein degradation pathways. Synonymous mutations in the *Maoa* gene can indeed impact gene expression regulation. Previous research has highlighted several epigenetic regulatory mechanisms affecting MAOAexpression and activity. For instance, the *Maoa* promoter’s critical regions directly control its expression, with promoter deficiency leading to a substantial reduction in *Maoa* activity in various human cell lines (Gupta et al., 2015). Methylation mechanisms can regulate MAOA-associated long noncoding RNAs (MAALIN), which, in turn, inhibit MAOA expression in the brain (Labonté et al., 2021). Additionally, Hdac6, a key player in misfolded protein degradation, can mediate the transport of misfolded proteins for aggregate degradation (Liu et al., 2016). The excessive translation speed caused by the GAC best codon may result in protein misfolding and subsequent *Maoa* degradation. PTBP3, a well-studied RNA-binding protein, is involved in pre-mRNA splicing and various aspects of mRNA metabolism, including polyadenylation, mRNA stability, and translation initiation (Romanelli et al., 2013). PTBP3 binds to *Maoa*’S 5’-UTR, promoting ribosome entry and translation. However, *Maoa* translation products are ultimately reduced due to ubiquitination-mediated degradation of RBBP6 and Hdac6.RAM22 plays a role in pre-mRNA splicing as part of activated spliceosomes, participating in the first step of pre-mRNA splicing. Zcchc8 is involved in monitoring and turnover of abnormal transcripts and noncoding RNAs. Yctd1 is a regulator of alternative splicing and recognizes N6-methyladenosine (m6A)-containing RNAs (Shima et al., 2017). These cleavage factors specifically bind to the WT sequence, ensuring mRNA activity and stability. On the other hand, the binding proteins involved in the KI sequence include RAMx2 from the RAMX family, which participates in pre-mRNA splicing but is slightly less crucial in mRNA cleavage than RAM22. Adar can alter mRNA translation by modifying amino acid sequences, splicing site recognition sequences, and RNA sequences involved in nuclease recognition. SLTM, when overexpressed, acts as a general transcription inhibitor, ultimately leading to apoptosis. These differences in binding proteins contribute to lower mRNA levels and reduced *Maoa* product levels in the KI sequences, which align with the findings from QPCR and WB experiments.

While DNA levels in each cell remain relatively constant, mRNA levels are subject to dynamic fluctuations, making RNA-seq a powerful tool for quickly determining changes in transcriptional activity. To investigate whether the synonymous mutation in the *Maoa* gene leads to differences in the expression of genes involved in transcriptional and translational regulation, RNA-seq was conducted on RNA extracted from mouse brain regions. The QPCR verification results indicated an upregulation in the expression of genes such as Drd1 and Dbh enzyme. These findings suggest that the midbrain limbic dopaminergic system, which can enhance dopaminergic signaling from the ventral tegmental area (VTA) to the ventral striatal nucleus accumbens (NAc), may be influenced by the *Maoa* gene and play a role in addiction. This enhancement in dopaminergic signaling is achieved by both increasing the excitatory input of dopaminergic neurons and inhibiting GABAergic neurons in the VTA region (Lüscher, 2016).

Additionally, KEGG classification enrichment analysis revealed that the Sf3a2 gene, involved in pre-mRNA splicing as part of the splicing factor SF3A complex, was downregulated. This decrease in mRNA processing reflects the instability of the KI-type gene mRNA. Furthermore, several translation-related genes, including those involved in ribosomal subunit composition, DNA methylation, shearing factors, protein folding, sorting, and degradation, exhibited differential expression in the PFC brain region. These results demonstrate that synonymous mutations can impact the expression and stability of the *Maoa* gene by regulating the upward and downward regulation of genes related to transcriptional and translational processes.

Our study provides valuable insights into the influence of coding region SNP synonymous mutations on epigenetic regulation, protein synthesis dysfunction, and dopamine system abnormalities. It sheds light on the cytoplasmic function of RNA-binding protein (RBP) in *Maoa* translation, offering a promising avenue for further research in this area. Using CRISPR/Cas9 technology to create a genetic mouse model that simulates the expression of the *Maoa* c.1409 T > C mutation in animals is a commendable approach to investigate potential epigenetic effects. Animal models are crucial for understanding the *in vivo* implications of genetic mutations. While Our study has made significant progress, there are indeed some limitations, such as the cell model of the gene mutation. Further experiments could delve deeper into the molecular mechanisms involved in how RBP proteins regulate specific processes related to transcription and translation. This would provide a more comprehensive understanding of the underlying mechanisms at play. Overall, Our research contributes to the growing body of knowledge on the impact of synonymous mutations on gene expression and protein function, particularly in the context of the dopamine system and related disorders. It also highlights the potential for future investigations in this field.

## Data availability statement

The datasets presented in this study can be found in online repositories. The names of the repository/repositories and accession number(s) can be found at: https://www.ncbi.nlm.nih.gov/genbank/, GSE237077.

## Ethics statement

The animal study was approved by the China National Tobacco Quality Supervision and Test Center, CTQTCSYXK- 2023003. The study was conducted in accordance with the local legislation and institutional requirements.

## Author contributions

KL: Data curation, Methodology, Writing – original draft. LF: Formal analysis, Validation, Visualization, Writing – original draft. HW: Supervision, Writing – review & editing. YT: Data curation, Writing – original draft. SZ: Writing – review & editing. QH: Supervision, Funding acquisition, Writing – original draft. FL: Writing – review & editing. HC: Funding acquisition, Writing – review & editing. HH: Data curation, Funding acquisition, Supervision, Writing – review & editing.
